# Meristem Plant Cells as a Sustainable Source of Redox Actives for Skin Rejuvenation

**DOI:** 10.3390/biom7020040

**Published:** 2017-05-12

**Authors:** Liudmila G. Korkina, Wolfgang Mayer, Chiara de Luca

**Affiliations:** 1 Centre of Innovative Biotechnological Investigations “NANOLAB”, 197/2 Vernadsky Pr., Moscow 119571, Russia; 2 Medena AG., Industriestrasse 16, CH-8910 Affoltern-am-Abis, Switzerland; wolfgang.mayer@medena.ch (W.M.); chiara.deluca@medena.ch (C.d.L.)

**Keywords:** cosmetics, environmental stress, meristem plant cells, plant metabolites, polyphenols, RNS, ROS, skin photoageing, skin rejuvenation, UV

## Abstract

Recently, aggressive advertisement claimed a “magic role” for plant stem cells in human skin rejuvenation. This review aims to shed light on the scientific background suggesting feasibility of using plant cells as a basis of anti-age cosmetics. When meristem cell cultures obtained from medicinal plants are exposed to appropriate elicitors/stressors (ultraviolet, ultrasound ultraviolet (UV), ultrasonic waves, microbial/insect metabolites, heavy metals, organic toxins, nutrient deprivation, etc.), a protective/adaptive response initiates the biosynthesis of secondary metabolites. Highly bioavailable and biocompatible to human cells, low-molecular weight plant secondary metabolites share structural/functional similarities with human non-protein regulatory hormones, neurotransmitters, pigments, polyamines, amino-/fatty acids. Their redox-regulated biosynthesis triggers in turn plant cell antioxidant and detoxification molecular mechanisms resembling human cell pathways. Easily isolated in relatively large quantities from contaminant-free cell cultures, plant metabolites target skin ageing mechanisms, above all redox imbalance. Perfect modulators of cutaneous oxidative state via direct/indirect antioxidant action, free radical scavenging, UV protection, and transition-metal chelation, they are ideal candidates to restore photochemical/redox/immune/metabolic barriers, gradually deteriorating in the ageing skin. The industrial production of plant meristem cell metabolites is toxicologically and ecologically sustainable for fully “biological” anti-age cosmetics.

## 1. Primary and Secondary Plant Metabolites: Biosynthesis and Impact on Plant Physiology

Two major classes of metabolites exist in plants. Primary metabolites are essential for plant survival and propagation (carbohydrates, proteins, amino acids, lipids, and fatty acids). Also, plants produce a great variety of other low-molecular weight organic compounds called secondary metabolites, which are not directly involved in the primary metabolic processes of growth and development. Secondary metabolites play multiple essential roles in normal plant physiology. They are mainly synthesised at need, and function primarily in adaptation to biotic and abiotic stresses. As for the biotic aggressions, these metabolites protect plants against pathogens such as virus, mycoplasma, bacteria, and fungi, against predators—both insect and mammal herbivores—rival species, and plant competitors [1,2]. Numerous secondary metabolites are synthesised to protect a host plant from abiotic stresses like sunlight (ultraviolet (UV), visible, and infrared), ozone, temperature changes, draught, salinity, and herbicides (reviewed in [3]). They provide capacity for adaptation to continuously changing environmental conditions as well as reproductive advantages attracting pollinators and seed dispersers, they serve as signalling molecules and hormones, and guarantee competitive benefits by the poisoning of rival species [4].

Secondary metabolites are derived from primary metabolites, first of all the amino acid phenylalanine, carbohydrates, and lipids, through methylation, hydroxylation, and glycosylation reactions. Up-to-date, several thousands of different secondary molecules have been identified in plants. By chemical structure, plant-derived secondary metabolites belong to three major classes: the terpens (isoprenoids, terpenoids), the phenylpropanoids and their “off-springs” polyphenols (flavonoids, tannins, glycosides, lignans, etc.), and the nitrogen-containing molecules (alkaloids and heterocyclic aromatics) [5].

The biosynthesis of plant polyphenols (flavonoids, isoflavonoids, coumarines, curcuminoids, and lignans) shares a common initial step—the deamination of phenylalanine to cinnamic acid, which is catalysed by phenylalanine ammonia lyase (PAL, Enzyme Catalogue 4-3.1-5), a family of enzymes responsive to different developmental and environmental stimuli. Several exogenous factors are known to affect PAL expression and activity, such as intense light, UV, ultrasonic (US) and gamma-ray irradiation, wounding, infections, heavy metals, and organic toxins [3]. Branch pathways lead to the synthesis of compounds with diverse defensive functions in plants, such as cell wall strengthening to prevent pathogen penetration (polyphenol polymers), wound repair (lignins and suberins), antimicrobial activity (furanocoumarins, pterocarpans, and phytoalexins), and alarm function (salycilic acid) [6]. Definite plant phenolics, for example scopoletin, quercetin, etc. are often converted by phenol oxidases and peroxidases into more reactive species to kill pathogens and eliminate photo-toxins [7,8]. The group of active plant metabolites could be considered as a primitive “innate immune system”, exerting either direct antimicrobial activity or providing chemotactic signals to both pathogenic and symbiotic microorganisms [8,9].

**Figure 1:**
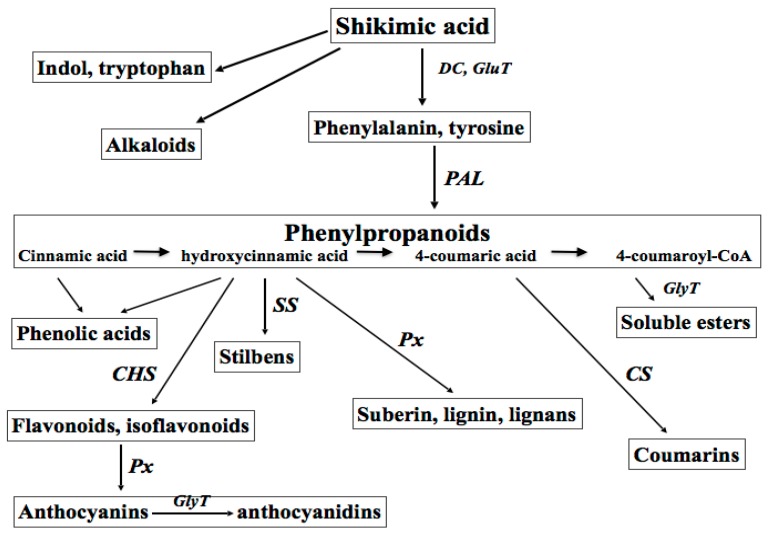
The super-family of plant secondary metabolites of actual and potential use in anti-age cosmetics. The parent molecule shikimic acid is transformed into phenylalanine. Inducible by a variety of biotic and abiotic stresses, the enzyme phenylalanine ammonia liase (PAL) is a key enzyme for polyphenol biosynthesis, having and cinnamic acid is the first product. Then, upon the action of different enzymes, such as oxidases, peroxidases, transferases, synthase, etc., numerous “off-springs” of the parent molecules are formed. DC: decarboxylase; GluT: glutamyl transferase; GlyT: glycosyl transferase; CS: coumarin synthase; SS: stilbene synthase; ChS: chalcon synthase; PPx: phenol peroxidases.

Plant secondary metabolites are among the most common biologically active components of food, spices, aromas, fragrances, propolis, wines, essential oils, beer, and of traditional medicine. Taking into account their numerous roles in plant physiology, these compounds have attracted great interest in the last decades, especially for medicinal use as natural antioxidants, UV screens, anticancer, chemo-preventive, anti-virus, anti-inflammatory, wound healing, and antibacterial agents [2,4,5,10]. Great interest in both natural and synthetic derivatives of plant metabolites has been recently shown by cosmetic and perfume industries. The classes of plant-derived polyphenols with a potential for cosmetic industries are shown in Figure 1.

## 2. Mechanisms of Secondary Plant Metabolites Interaction with Human Cells

All primary molecular targets of plant secondary metabolites in the human body may be specific or nonspecific. Usually, specific targets are the active sites of enzymes, the binding sites of receptors, or transcriptional factors. Due to the high binding affinity, plant-derived metabolites are able to compete with endogenous ligands, thus inhibiting or inducing the corresponding metabolic or signal transduction pathways. For example, some plant metabolites are chemically similar to phenols which are biologically active in humans, such as L-tyrosine, adrenalin, noradrenalin, L-3,4-dihydroxyphenylalanine (L-DOPA), thyroxin, and estrogens. They could either compete with or mimic these molecules in the metabolic and/or signal transduction pathways, affecting significantly the functions exerted by these amino acids, hormones or neurotransmitters. It is demonstrated that plants can biosynthesise secondary metabolites such as indoleamines, melatonin, and serotonin [11,12,13,14], the polyamines putrescine, spermin, and spermidine [15], and L-DOPA [3], all of which are well known for their essential functions in human organism. The regulatory functions in development and senescence [15], as well as the protective and adaptive roles of these secondary metabolites for plant cells exposed to cold, light of different intensity and spectrum, water and soil pollutants, nutrient deficit, etc., have been extensively evaluated [3,14].

A variety of biological effects of secondary metabolites are carried out through the non-specific interaction with various targets, from proteins to small molecules and ions. In this sense, the widest attention has been devoted to the inhibition of free radical-driven reactions in biological systems by low-molecular weight plant molecules, through either chain-breaking or free radical scavenging, or/and transition-metal chelating mechanisms (direct antioxidant action), as widely published elsewhere.

Recently, the essential role of secondary plant metabolites in the induction or suppression of gene expression in human cells has been recognised. Being mainly polycyclic aromatic molecules, these metabolites stimulate human genes responsible for their detoxification/metabolism (phase I and II metabolic enzymes) and their fast excretion from the organism. Furthermore, the genes encoding enzymes detoxifying active oxygen and nitrogen species, such as Mn-superoxide dismutase, catalase, and glutathione-metabolising enzymes, are activated as well, because these potentially toxic species are formed in the course of the metabolic transformation of plant-derived molecules. This mechanism is a basis for a long-lasting and efficient indirect antioxidant action of plant metabolites. The molecular pathways underlying the expression of detoxifying and antioxidant genes have been intensely investigated during the last decade. In accord with a current concept [16,17,18,19], the very first sensor of secondary plant metabolites in human cells is the aryl hydrocarbon receptor (AhR), which activates the downstream genes of cytochrome P450 superfamily, phase I bio-transforming enzymes, which in turn hydroxylate polyaromatic molecules to more water-soluble metabolites. These metabolites become substrates for the phase II conjugating enzymes, such as glutathione-S-transferases (GSTs), UDP-glucoronyl transferases (UGTs), catechol-*O*-methyl transferase (COMT), N-acetyl-transferases (NATs) and others. The metabolites conjugated with sulfhydryl, acetyl, methyl, and other groups are then easily excreted from the body.

The transcriptional response to secondary plant metabolites in human cells is typically mediated by the cis-acting antioxidant response element (ARE), found in the promoter of the encoding genes for diverse products such as several GSTs, metalloproteinases, NADPH: quinone oxidoreductase 1, UGTs, gamma-glutamate cysteine ligase, heme oxygenase 1, and peroxiredoxin VI. The major ARE-binding transcription factor is nuclear factor-erythroid 2-related factor 2 (Nrf2) which, through a heteromeric interaction with the small Maf proteins, binds ARE and initiates the de novo expression of detoxifying enzymes [18,19,20,21,22,23].

**Figure 2:**
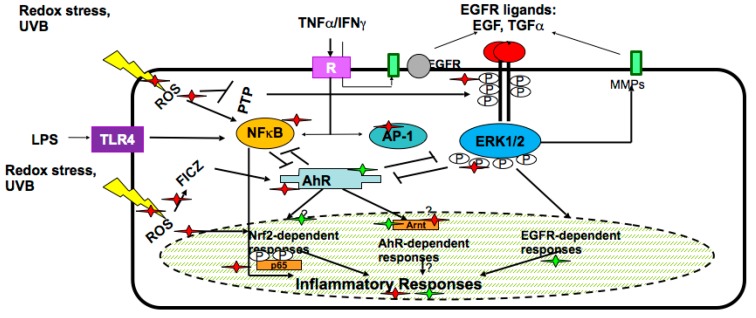
Metabolic and inflammatory pathways in keratinocytes affected by biotechnologically produced plant secondary metabolites. Secondary plant metabolites, mainly polyphenolics, may enhance (green asterisks) or inhibit (red asterisks) metabolic and inflammatory responses of keratinocytes to abiotic stresses (ultraviolet (UV) irradiation or reactive oxygen species (ROS) stress) or biotic signals—inflammatory cytokines, (tumor necrosis factor alpha (TNF-α) or interferon gamma (IFN-γ)), bacterial lipopolysaccharides (LPS) or ligands for epidermal growth factor receptor (EGFR).

In the skin keratinocytes, plant secondary metabolites target epidermal growth factor receptor (EGFR)-linked pathway, interconnected with the two previously described metabolic systems regulated by AhR and Nrf2 transcription factors. In the complex network responsive to plant-derived molecules, there exist several pathways/regulatory factors such as nuclear factor kappaB (Nf-κB), mitogen-activated kinases (MAPKs), extracellular responsive kinases (ERKs), etc., which are involved in the inflammatory responses of keratinocytes to external stimuli [4,19,21]. The molecules and pathways in human skin keratinocytes affected by secondary plant metabolites are numerous and they are schematically presented in Figure 2.

## 3. Limitations of the Industrial Development of Health Products Based on Plant Secondary Metabolites

The industrial development and the commercial utilisation of secondary plant metabolites in health products are restrained by a number of factors: (1) limited availability from natural sources; (2) seasonal variations in plant harvesting; (3) contamination of plant raw material with environmental pollutants and toxins (e.g., heavy metals, oil- and gas-derived toxic hydrocarbons, pesticides, aflatoxins, mycotoxins, and other organic and non-organic hazardous compounds); (4) complex and expensive procedures for the extraction and purification of the active substances from the grown plant tissues; (5) poor standardisation of the final product due to unavoidable variations in soil, water, weather conditions, and fertilisers used for the plant growth.

To overcome some of these limitations, there is now growing interest in the use of biotechnological approaches to produce plant-derived active substances using non-genetically modified plant cell or plant tissue cultures. Moreover, as the demand for secondary metabolites derived from medicinal plants is growing steadily at a very fast pace, the survival of these plants is increasingly threatened, as many of them have become extinguishing species. Therefore, in search for alternative methods to obtain the desired natural compounds, plant cell culture technologies have been developing as a valuable source of high-quality plant-based medicinal/cosmetic ingredients, as well as a tool for elucidating the bio-synthetic pathways of plant secondary metabolism [24,25]. Recent advances in plant cell cultivation, including high-density suspension cultivation and continuous culture, manipulation of environmental factors and of the supply of precursors and oxygen, elimination of final metabolites, challenge by appropriate elicitors, and selected cell culture scale-up have provided breakthrough and hopes for a highly standardised production of secondary metabolites of pharmaceutical and cosmetic value [26]. The emerging approaches based on secondary metabolism regulation in plant cell cultures by microRNAs open huge perspectives in the induction and stable production of valuable substances [27]. Further developments in the bioreactor cultivation processes, and in the metabolic engineering of plant cells for metabolite production are expected in the near future [28,29,30,31,32].

## 4. Meristem Plant Cells as a Source of Secondary Metabolites

**Figure 3:**
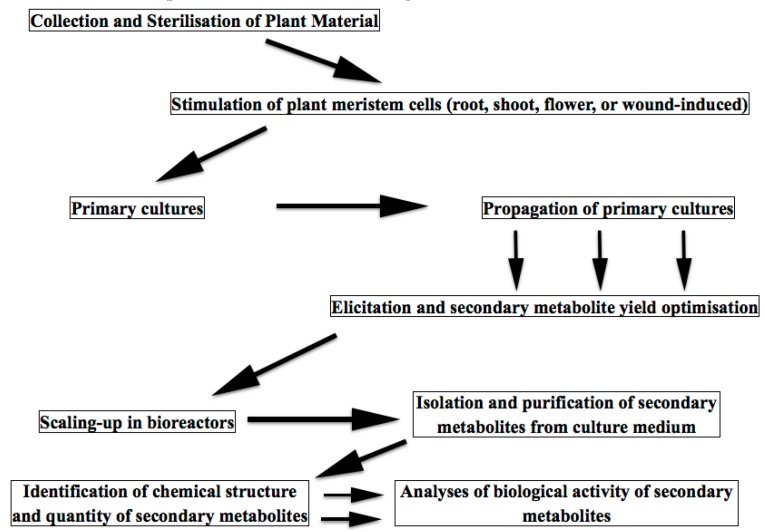
Scheme of secondary metabolite production by meristem plant cells.Meristem cells are stimulated and collected from shoots, roots, flowers or leaves of grown plant under sterile conditions. Cells are cultivated and up-scaled to industrial volumes in nutrient medium and exposed to biotic or abiotic elicitors. Medium containing secondary metabolites released from cultured plant cells is collected, secondary metabolites are isolated and analysed both qualitatively and quantitatively. Biological activity of secondary metabolites is determined in silico, in vitro in acellular and cell-containing systems, organ cultures, and in vivo (on healthy volunteers or in clinical trials). In vivo animal studies of cosmetic ingredients are prohibited by the international law.

Plant in vitro technologies have been known for more than a century. At some point, pharmaceutical and cosmetic industries recalled a 19th century discovery about the cultivation of meristem plant cells. By a very simple procedure, the meristem cells are obtained either from plant shoots or flowers or roots and are cultivated under defined conditions, giving rise of plant cell conglomerates or suspensions having lost the morphological features of the parent plant [3,6,30]. Meristem cells play essential and multiple roles in plant physiology participating in plant growth, regeneration, flowering, leaf and branch sprouting, wound healing, etc. Meristem cell cultivation has been developed for various purposes, such as re-production of different cultivars, genetic modification of plant species, preservation of extinct species, production of virus-free plants, assessment of plant metabolism and physiology, etc. Only since 15–20 years ago, these plant cell/tissue cultures have become increasingly attractive as cost-effective alternatives to classical approaches for a sustainable high-yield production of plant-derived molecules (so-called “green cell factories” concept), because of their several advantages [33]. Of utmost importance, the plant cell/tissue culture is the only ethical and environmentally sustainable way of producing high-value metabolites from rare and/or threatened plants [34]. Schematically, a biotechnological process for meristem cell metabolites production is shown in Figure 3.

The secondary metabolites produced in plant cell/tissue cultures are easily available in the cultivation medium, since they are able to cross the outer cellular membrane by exudation and accumulate either in the form of liquid medium solutions, or attached to absorbents introduced into the cultural environment [35]. The biosynthesis of water-soluble phenolics, such as rosmarinic acids, cinnamic acid, caffeic acid, and coumarins in the callus cultures of *Salvia miltiorrhiza* Bunge could be elicited by methyl jasmonate, salicylic acid, abscisic acid, polyamines, metal ions, hydrogen peroxide (H₂O₂), UV-B radiation, and yeasts through the subsequent induction of the genes of PAL, cinnamic acid 4-hydroxylase (C4H), 4-coumarate-CoA ligase (4CL), tyrosine aminotransferase (TAT), 4-hydroxyphenylpyruvate reductase (HPPR), 4-hydroxyphenylpyruvated dioxygenase (HPPD), and hydroxycinnamoyl-CoA:hydroxyphenyllactate hydroxycinnamoyl transferase-like (RAS-like) [36]. Plant tissue/organ cultures derived from *Syringa vulgaris* L., *Buddeleia davidii* Franch., and *Harpagophytum procumbens* DC. (exMeisn.) are in turn valuable and sustainable sources of verbascoside, a glycosylated phenylpropanoid well characterised for a wide range of health effects towards skin cells in vitro and in vivo [30,37,38,39].

Isoflavonoids are widely used in cosmetic preparations with anti-ageing claims. The production of the isoflavones genistein, daizein, and isoprunetin, as well as of their substituted derivatives 5-methylgenistein and *O*-glucosyl-isoflavones possessing remarkable estrogenic properties [40,41], has been elicited in suspension cultures of *Genista tinctoria* L. by a low-level US treatment [42]. While high-intensity US waves damage cells and lead to enzyme denaturation, low-level US application is able to shift cellular metabolism towards the increased production of secondary metabolites, for example the ginsenoside saponins of *Panax ginseng* (Meyer) [43] and shikonins of *Lithospermum erythrorhizon* Sieb et. Zucc. cells [44], to combat with the external physical stress. Thus, US irradiation of *Panax ginseng* cell cultures induced the expression of polyphenol oxidase, peroxidase, and PAL, and the production of polyphenols [45]. The exposure of *Genista tinctoria* cell cultures to electrical field or AgNO_3_ led to highly increased levels of isoflavones, such as genistein and daidzein, in the cultural medium [46].

Stilbens like resveratrol and its glycosylated derivatives as well as anthocyanines exert antioxidant, anti-inflammatory, anti-UV, and anti-ageing actions in vitro, in different skin cell-containing systems [37,47,48,49], in vivo in animal experiments focused on skin structure and functions [50,51], and ex vivo in human clinical studies where they were applied topically [52,53]. Consequently, these plant-derived polyphenolics have been included in numerous cosmetic preparations available on the market. Due to a fast growing demand for these substances as active ingredients of cosmetic preparations, their industrial production has been established, in *Vitis vinifera* L. cell suspension culture elicited by indanoyl-isoleucine, *N*-linolenoyl-l-glutamine, insect saliva, and low micromolar concentrations of heavy metals, such as Co, Cd, and Ag [54,55]. The thorough elucidation of their bio-synthesis confirmed that resveratrol and glycosylated resveratrol pathways were activated as an early stress response of *Vitis vinifera* cells to biotic and abiotic stressors, while polymerisation of polyphenol monomers to anthocyanines was a delayed process.

Recent research [56] has shown that jasmonic acid, an unsaturated fatty acid of plant origin, and its methyl esters (oxylipins or jasmonates), being considered classical elicitors of secondary metabolism in plant cell cultures, also induce the biosynthesis of saturated and unsaturated (first of all, alpha-linolenic acid (C18:3ω3)) fatty acids in *Catharanthus roseus* (L.) G. Don cell suspension cultures, as a primary stress response. This peculiar activation of fatty acid biosynthesis in plant cultures may become a valuable source of these highly requested ingredients for anti-age cosmetics.

## 5. Redox Regulation of Secondary Metabolite Synthesis in Plant Cell Cultures

It is common knowledge that the induction of secondary metabolite synthesis is a primary defence response of plant cells to various stresses [4,57]. The molecular pathway starts from specific receptors on plant cells to recognise UV light and respond to its challenge [58,59]. Thus, UV-B-induced defensive response primarily activates the phenylpropanoid synthesis, phenylpropanoid transformation into flavonoids, and flavonoid glycosylation [60]. Accumulation of certain flavonoids was observed in cultivated parsley cells irradiated by UV-B [61]. The physiological significance of such enhanced yields has been attributed to the UV-B-absorbing capacity of flavonoids and to their reactive oxygen species (ROS)—scavenging activity [62,63,64], significantly reducing the risk of UV-B-induced and free radical-mediated damage to plant cells. It has been reported that UV-B irradiated cultured plant cells simultaneously accumulated flavonoids and nitric oxide (NO) [65,66,67]. Keeping in mind that NO is an important signalling molecule mediating the defence response of both plant and animal cells to UV-B [68,69], the key role of NO in the up-regulation of synthesis of baicalin, a glycosylated bioactive flavonoid, in meristem *Scutellaria baicalensis* Georgi cells has been shown elsewhere [70]. However, the source of NO, as well as its metabolic target in the phenylpropanoid synthetic pathway remain poorly understood. It is assumed that in plant cells NO could be synthesised enzymatically by nitric oxide synthase (NOS) or NOS-like enzymes [70]. Another source of NO could be an inorganic nitrogen pathway. Several recent data support the existence of NOS-like enzymes which are sensitive to pharmacological inhibitors of mammalian NOS [71,72]. Moreover, NOS gene has been identified in *Arabidopsis* sp. plant cells, regulating their growth and hormonal activity [73]. Nitric oxide is being recently recognised as a key regulator of diverse plant cellular processes. Major mediators of NO action towards plant cells and tissues are *S*-nitrosylated proteins, in which NO is bound to a cysteine forming *S*-nitrosothiol. For example, *S*-nitrosoglutathione may represent an important endogenous source of NO, and these two redox-active molecules play pivotal roles in plant immunity and secondary metabolite biosynthesis [74].

Salicylic acid (SA), synthesised in practically all parts of plants, has been since long time recognised as a growth hormone regulating shoots and roots growth in higher plants [75,76]. Then, several new functions of SA have been discovered, one of them being to provide an alarm signal for the induction of local and systemic defensive responses in the plant exposed to infections [75,77], organic and inorganic toxins [78], or UV-B irradiation [3]. Recently, SA is widely used as an elicitor of secondary metabolite biosynthesis in plant cultures. For example, exogenous applications of SA induced the biosynthesis of coumarins in *Matricaria chamomilla* L. [79], taxane in *Taxus chinensis* Rehder [80], saponins in ginseng [81], phenolic acids including rosmarinic acid in *Salvia miltiorrhiza* [66,82], and artemisin in *Artemisia annua* L. cultures [83]. A number of recent mechanistic studies have been carried out to elucidate connective pathways between SA challenge and the induction of metabolic pathways of secondary metabolites in plant cultures [82]. It appears that the H_2_O_2_ produced by NADPH-oxidases of plant cells challenged by SA is an essential mediator of signal transduction from SA to secondary metabolism [82,84]. According to one hypothesis, SA binds to genes encoding proteins with catalase-like activity in different plant cells [77], thus inhibiting the enzyme. Catalase deficiency inevitably leads to sharply and highly increased levels of H_2_O_2_ that, in its turn, initiates the synthesis of secondary metabolites with antioxidant and redox-regulating properties, to reduce a risk of oxidative damage [77,82]. Certain data argued that after a primary event of catalase inhibition by SA, later metabolic changes may bring an increase in antioxidant enzymes, such as catalase and superoxide dismutase, in plant cells. However, the mechanism of secondary induction of antioxidant enzymes in plant cells remains obscure. Lately, H_2_O_2_-dependent signal transduction has been implicated in the induction of the secondary metabolism initiating enzyme PAL first of all, and in the phenylpropanoid and glycosylated polyphenol synthesis in plant cell cultures exposed to a variety of elicitors, such as heavy metals, UV irradiation, lipid elicitors (jasmonic acid and jasminoids), microbial polysaccharides, etc. [36,82,85,86,87]. In general, ROS are ranked as the most relevant signal transduction agents ubiquitously present in plant cells [88,89,90,91].

The US-induced secondary metabolism observed in plant cell cultures could be regulated by low-level ROS, which have been shown to be produced in sonicated cell-free solutions [92,93] and in cells upon exposure to moderate U.S. [94]. This effect has become a background for the sonodynamic therapy of tumours [95,96]. Thus, a 2 min sonication of *Taxus* cell cultures induced rapid and dose-dependent NO production, followed by induction of taxol biosynthesis [97]. UV and US treatment of peanut cultures resulted in increased α-resveratrol (2.64–4.40 μg/g), piceid (polydatin, glycosylated resveratrol, resveratrol 3-beta-mono-*D*-glucoside), and total stilbenes, by the NO-dependent stimulation of their biosynthesis [98].

ROS are rapidly produced in plant cells as a ubiquitous early response to pathogens (similar to the oxidative burst in human cells) and abiotic stressors. While plant and human cells alike, being challenged by pathogens, rely on cytoplasmic membrane-bound NADPH-oxidase as a source of superoxide, plant cells have, in addition, chloroplasts- and peroxisomes-located sources of ROS (xanthine oxidase, oxalate oxidase, and peroxidases), sensoring mainly abiotic stresses [99]. ROS initiate the development of resistance mechanisms such as the biosynthesis of secondary polyphenols with antioxidant and free radical scavenging properties.

State-of-the art knowledge provides therefore clear-cut evidences on the key regulatory functions of relatively stable ROS and reactive nitrogen species (RNS), such as hydrogen peroxide and NO, in signal transduction from the elicitor/environmental stressor to the biosynthetic chain of enzymes involved in the production of secondary metabolites.

Furthermore, we dare hypothesising that cultured plant cells may share some mechanisms of endogenous antioxidant and metabolic defence with mammalian and microbial cells. Mammalian cells are mainly armed by the Nrf2-regulated defensive system to protect themselves from damaging oxidants and electrophiles [19,100]. Molecular mechanisms of the adaptive response to oxidative stress in bacteria are complex and similar to those of mammalian cells. They are controlled by key transcriptional regulators, such as OxyR (analogous to Nrf2 in mammalians), and peroxide operons regulator (PerR) sensing hydrogen peroxide when it oxidises thiolates or iron moieties, respectively [101,102,103], while redox-sensitive transcriptional activators SoxR and SoxS seem to control levels of redox-cycling compounds [104]. Microbes successfully exploit this defence against oxidative stress created by drug exposure, thus becoming resistant to multiple antibiotics [105,106].

Due to the constant challenge of the highly oxidative/hazardous environment, plants seem to activate, as early as within the first 8 h, mechanism(s) of systemic resistance based on synthesised-upon-request polyphenols with strong UV-screen, antioxidant, and redox-damage restricting features. The biosynthesis starts from the stimulation of PAL as a key enzyme, followed by methylation, glycosylation, oxidation, and polymerisation of primary phenylpropanoids. In a protective cycle, synthesised polyphenolics could induce Nrf2-like mechanism leading to an up-to-48 h delayed induction of antioxidant enzymes [66]. However, major players and pathways are still to be discovered. This additional plant defence guarantees survival, resistance to harsh climatic conditions, and advantages versus growth competitors.

## 6. Universal Protection Provided by Secondary Plant Metabolites to Plant and Mammalian Cells

The protective properties of plant secondary metabolites against solar light of broad spectral range from UV irradiation to visible and infrared light, ROS, RNS, heavy metals, and microbiota are greatly similar for plant and mammalian cells. This possibility of universal defence is provided by peculiar chemical structures (chromophores) that enable the absorbance of solar light energy and either dissipate it in thermal reactions (sunscreen properties), or convert it into chemical reactions (photochemical properties). In the presence of molecular oxygen, such photochemical reactions result in a ROS production. A great majority of secondary plant metabolites belong to the redox-active compounds which, depending on their intrinsic redox potential and environment, might exert opposite ROS/RNS scavenging or promoting actions.

### 6.1. Interaction with UV

Biotechnologically produced meristem cell-derived secondary metabolites are extremely efficient in the protection of human skin keratinocytes in culture and human skin in vivo from the damaging effects of solar UV irradiation [37,107,108,109,110]. With their molecular structure consisting of condensed aromatic 5- and 6-carbon rings with multiple OH-groups, they effectively absorb UV-A and UV-B irradiation (sunscreen properties), although without promoting further photo-chemical reactions; therefore, they have been considered superior to classical synthetic sunscreens [111]. The presence of the glycosyl moiety provides increased photo-stability, hence many of glycosylated metabolites are not destructed by UV-A light. In general, secondary plant metabolites may also be able to modify skin-UV interactions at several crucial points: (a) absorbing UV-A + UV-B light (screen action); (b) interrupting UV-induced free radical reactions in skin cells and extracellular matrix (scavenging and direct antioxidant chain-breaking effects); (c) protecting endogenous skin-surface lipid antioxidants, such as alpha-tocopherol, coenzyme Q_10_, and squalene (antioxidant rescue action); (d) inducing endogenous antioxidant systems in keratinocytes and fibroblasts (indirect antioxidant effects); (e) attenuating inflammatory responses in keratinocytes (anti-inflammatory action via several molecular pathways in keratinocytes) (Figure 2); (f) modulating excessive metabolic and proliferative UV-induced stress responses (anti-stress effects); and (g) attenuating UV-induced immune suppression (immuno-modulation) [38,62,112,113]. Different meristem cell metabolites exert differential actions as preventive, sun-screen, or post-sun curative substances [107]. Meristem cell-derived active substances have proven to be extremely effective in the protection of human skin fibroblasts and blood vessel endothelial cells against cell damage derived from UV irradiation and bacterial exposure [38,107]. Human keratinocytes exposed to pro-inflammatory cytokines or bacterial lipopolysaccharides produced greatly decreased amounts of deleterious cytokines and reactive oxygen species in the presence of active substances from meristem plant cells [38]. Their protective action was comparable with that of topical corticosteroids and non-steroidal anti-inflammatory drugs [39,113].

### 6.2. Free Radical Scavenging and Antioxidant Properties

Secondary plant metabolites, polyphenols and their derivatives, depending on their chemical structure, one-electron redox potential, and the redox properties of the environment, may in particular exert either free radical scavenging/antioxidant or free radical generating/pro-oxidant action [5]. Numerous original papers and reviews consider direct free radical scavenging/antioxidant effects of various plant-derived polyphenols as a solid basis for their anti-ageing, cytoprotective, and chemopreventive effects [5,19,114,115]. At the same time, the free radical generating and pro-oxidant properties of polyphenols underlie their ability to induce endogenous antioxidant enzymes (superoxide dismutase, glutathione peroxidase, gamma-glutamylcysteine synthase) through Nrf2 mechanism (indirect antioxidant action) or phase II metabolic enzymes (glutathione *S*-transferase, hemoxygenase-1, NADPH quinone oxidoreductase), providing a detoxifying action through the bio-transformation of secondary plant molecules into low-toxicity and easily excreted substances [4,19,100].

Plant cell cultures exposed to appropriate elicitors appear to be a unique and valuable source of low molecular weight modulators of the oxidative state (targeted free radical scavengers, direct and indirect antioxidants) in biological systems. These low molecular agents are bioavailable and biocompatible with human skin cells, hence they could be considered as ideal modulators of cutaneous oxidative state. For example, grapevine berries (*Vitis vinifera*) are commonly considered as a valuable source of strong free radical scavengers and direct antioxidants—phenolic acids, catechins, and anthocyanins like cyanidin 3-glucoside, peonidin 3-glucoside, malvidin 3-glucoside, cyanidin 3-*p* coumaroyl glucoside, etc. [116,117,118]. Callus suspension cultures of *Vitis vinifera* were elicited by indanoyl-isoleucine, *N*-linolenoyl-l-glutamine, insect saliva, and malonyl coenzyme A, all of them mimicking environmental pathogens, which provoked the induction of the phenylpropanoid biosynthetic pathway. This resulted into highly increased de novo synthesis and release of phenolic acids and resveratrol derivatives into the culture medium [119]. Similarly, various stressors applied to plant cell cultures, such as UV and blue light, high intensity light, wounding, pathogen attack, drought, sugar, and nutrient deficiency led to anthocyanin accumulation [120], and induced phenylpropanoid biosynthesis [1]. Phenylpropanoids are themselves strong antioxidants/free radical scavengers/metal chelators. They are also parent molecules for practically all known plant-derived polyphenols with free radical scavenging and antioxidant properties [2,34]. Light-grown suspension cultures of *Artemisia absinthium* L. exhibited gradually increasing antioxidant activity, which correlated with increased total phenolics and total secondary metabolites production [121]. Metabolomic analysis has revealed that the accumulation of specific secondary metabolites, namely, glycosylated phenylpropanoids (echinacoside, verbascoside, and related molecules) and caffeic acid conjugates with tartaric, quinic, and hexaric acids in cultured *Echinacea angustifolia* DC. cells was highly increased by cell culture illumination, while levels of rhamnosylated derivatives were significantly reduced [122]. Results suggested that the metabolic profile of secondary metabolites can be manipulated and optimised by controlling simple environmental variables, such as illumination. Four phenylpropanoid glycosides from plant cell cultures—verbascoside, forsythoside, echinacoside, and campneoside—proved to possess similar strong antioxidant and radical-scavenging activities as determined by diphenylpicrylhydrazyl (DPPH) assay [123]. Two of them, forsythoside and echinacoside, were activators of Nrf2, the nuclear transcription factor regulating antioxidant, cytoprotective, and detoxifying enzymes in HaCaT cells. Simultaneously, these substances reduced nuclear protein levels of the transcriptional regulator protein encoded by the *BACH1* gene, a repressor of the antioxidant response element [123]. Verbascoside from *Buddeleia davidii* meristem cells and its semi-synthetic acyl derivative were shown to be effective direct antioxidants and free radical scavengers feasible for dermo-cosmetic and pharmaceutical topical formulations [124]. *Vitis vinifera* cells grown on a medium enriched with ammonium nitrate, an important nitrogen source for plant growth and development, produced phenolics and resveratrol in amounts 15,9-fold and 5,6-fold higher than those grown on a conventional medium. Antioxidant activity estimated by the DPPH and 2,2’-azino-bis(3-ethylbenzothiazoline-6-sulphonic acid cation (ABTS^+^) assays positively correlated with the contents of phenolics and resveratrol [125]. Panduratin A, a flavonoid possessing remarkable antioxidant, anti-inflammatory, anti-cancer, and anti-viral properties, was produced in large amounts by cell cultures of *Boesenberia rotunda* (L.) Mansf. grown in a medium enriched with phenylalanine, as a precursor of flavonoid biosynthesis. Transcriptome sequencing and digital gene expression analyses revealed that in the phenylpropanoid pathway leading to panduratin A biosynthesis, phenylalanine ammonia-lyase, 4-coumaroyl:coenzyme A ligase, and chalcone synthase were greatly up-regulated [126]. Shikonins are commercially attractive secondary metabolites, known for their antioxidant, anti-tumour, anti-microbial, and insecticidal effects. The major sources for industrial production of shikonin and its derivatives have been identified in the *Lithospermum*, *Arnebia*, *Alkanna*, *Anchusa*, *Echium*, and *Onosma* greatly endangered species. The recent progress in shikonin biotechnological production with optimal cell culture conditions, proper elicitation, in situ product removal, and metabolic engineering allowed to develop continuos, reproducible, and cost-effective systems to provide pharmaceutical, cosmetic, and food industries with these active substances [127]. The pharmaceutically and cosmetologically important silymarin, a free radical scavenger, antioxidant, and UV-B protector, is now produced employing *Silybum marianum* L. Gaert tissue cultures. This plant has been widely recognised as having a high biosynthetic capacity to produce the metabolite of interest. The efficient silymarin-producing cell line has been established, elicitors and culture conditions have been optimised [128].

Isoflavonols with estrogenic functions (phytoestrogens) and direct antioxidant capacity are highly requested for anti-age cosmetics. Enhanced daidzin (7-*O*-glucoside of daidzein) production was achieved by eliciting hairy root cultures of *Psoralea corylifolia* L. by salicylic and jasmonic acids [129].

Melatonin, a neurohormone produced by the pineal gland, has recently been discovered to exist also in the plant kingdom [11]. Elevated levels of melatonin seem to be involved in the intrinsic protection from water and soil pollutants. Constitutionally high contents of melatonin are found in acquatic plants, for example in the water hyacinth, which is extremely tolerant to organic toxins and heavy metals [130]. Melatonin is regarded as a broad spectrum antioxidant protective for human tissues and organs, and skin in particular [19,131]. Recently, phytomelatonin could be produced in industrial quantities in the cultures of acquatic plants [11]. Another example is the indoleamine serotonin, which is a neurohormone in vertebrates and a stress protector in plants. Phytoserotonin is also believed to scavenge reactive oxygen species, thus delaying the process of senescence, and protecting the young reproductive tissues of the plant from abiotic stresses, such as cold [13].

### 6.3. Heavy Metal Chelation

Flavonoids, phenylpropanoids, and their glycosylated derivatives with two adjacent -OH groups in any of aromatic rings can bind transition metals in redox inactive forms [132]. A plant cell line derived from *Ajuga reptans* was challenged to produce teupolioside (TP), a phenylethanoid glycoside with multiple biological actions. These TP-producing meristem cells were extremely effective in scavenging oxygen free radicals and peroxynitrite. and in the chelation of several transition metals, such as ferric, ferrous, copper, and nickel [4]. Mainly due to its chelating and antioxidant properties, TP from *Ajuga reptans* L. meristem cells exerted an inhibitory action towards superoxide-producing enzymes, lipoxygenase, tyrosinase, and 5-alpha-reductase. Since all these enzymes play a key role in the pathogenesis of certain skin disorders, namely inflammation, acne, hair loss, allergy to nickel, and hyper-pigmentation, cosmeceuticals and dermatological compositions containing TP have been developed and commercialised.

Quite a number of studies have shown that cadmium, a non-essential heavy metal released into the biosphere by various anthropic activities, possesses high toxicity potential towards human beings, plants and animals. Humans are exposed to Cd through diet, polluted air, and cigarette smoke [133]. Exposure to this heavy metal is currently associated with increased risk of vascular and smooth muscle pathologies as well as with skin ageing [134]. The mechanisms of Cd-related biological damage are thought to be tightly connected to oxidative stress, which arises through excessive ROS and RNS and down-regulated endogenous antioxidant systems, first of all the altered glutathione defence [3,133,135]. While in plants the first line of defence entraps Cd extracellularly by means of specific thiol-rich proteins phytochelatins and frustulins [136,137], in human cells metallothionines and glutathione synthesis are primarily induced to bind Cd into the redox inactive complexes. Then, the induction of antioxidant enzymes through Nrf2 pathway occurs, that prevents endoplasmic reticulum stress, mitophagy, and crucial metabolic alterations [138]. This endogenous Cd chelation seems to be insufficient to prevent/alleviate biological damage, hence preventive and therapeutic strategies based on natural non-toxic chelators and antioxidants have been developed. A dietary approach relying on edible plants and phytochemicals has been gaining grounds [139]. A wide range of plant antioxidants and extracts of black and green tea, *Allium sativum* L., *Zingiber officinale* Roscoe, *Physalis peruviana* L., *Aronia melanocarpa* (Michx.) Elliott and other plants have been shown to prevent Cd-induced oxidative stress and toxicity [140]. Of interest, suspension cultures of *Vitis vinifera* cells reacted to CdCl_2_ challenge with the induction of flavanol, flavonol, trans-resveratrol, and tocopherols biosynthesis, while their growth potential was suppressed by the same metal at high concentrations [141]. As it seems to be a self-sacrificing protective/adaptive strategy evolved in plants exposed to toxic metals, this strategic line has been exploited in an attempt to produce plant-derived metal chelators, mainly, flavonoids in plant cell cultures elicited by toxic heavy metals [3,35,46,55]. Metal ions such as lanthanum, europium, silver, copper, cobalt, zinc, and cadmium are able to up-regulate the biosynthesis of secondary metabolites with metal chelating properties as polyamines, anthocyanins, betacyanins, and scopolamine [3], while Ni ions and other trace metals inhibit the activity of PAL [142].

### 6.4. Effects on Endogenous Defensive Mechanisms in Human Cells

Secondary plant metabolites could be viewed as excellent stimulators of basic endogenous mechanisms of human skin protection against various stresses: redox, metabolic, UV-induced, and toxicity stress as well as infections. With regards to skin protection, plant-derived actives primarily regulate its function as redox generator/barrier providing intra-cutaneous homeostasis [19]. The physiological roles of low levels of ROS, RNS, and reactive lipid species are numerous as they act as primary intracellular signal transduction molecules; they are indispensable effectors in the homeostatic pathways leading to cell proliferation, differentiation, senescence and death; they serve as mediators of immune-inflammatory responses to external and internal stresses. Their impact to metabolic signalling has been under investigation during last few years, and a vast body of evidence confirms that they possess anti-bacterial, anti-viral, and anti-tumour potentials [20,143,144,145].

As a master switch of the cellular redox homeostasis, the cap’n’collar basic leucine zipper (CNC-bZip) transcription factor Nrf2 is considered a critical player in a complex machinery of intracellular ROS/RNS production and utilisation [146]. Following exposure to oxidants or electrophiles, the cytoplasmically located Nrf2 moves to the nucleus, where it binds to ARE in the upstream regulatory regions of genes encoding detoxification and antioxidant enzymes. Enhanced transcription of these genes follows [147,148]. Many secondary metabolites for example, phenylpropanoids, isothiocyanates, flavonoids, stilbenes, and indoleamines induce the dissociation of Nrf2 from Kelch-like ECH-associated protein 1 encoded by the *Kelch* 1 gene (Keap1), followed by nuclear translocation of the nuclear factor, and the expression of ARE-regulated cytoprotective, metabolic, and antioxidant genes [148].

### 6.5. Interaction with Skin Microbiota

In higher plants, a number of secondary metabolites with anti-microbial properties are synthesised when plants are infected with bacteria, viruses, mycoplasma, lichen, etc. [1,120,149]. For example, stilbenes, such as resveratrol and its derivatives, furanocoumarins, pterocarpan, and isoflavonoids are known as anti-bacterial and anti-fungal phytoalexins fighting plant infections [2,4,150].

Human skin, like plants, is in direct and constant contact with environmental microorganisms. Normally, skin harbours a plethora of different groups of symbiotic microbial agents (bacteria, viruses, fungi, and protozoa) organised in a community called human skin microbiota. The resident skin microbiota interacts with other microbes, with skin cells, and with the local cutaneous immune system in multiple ways that determine risk of skin diseases and accelerated ageing [151,152]. Epidermal keratinocytes continuously release antibacterial substances to keep local microbiota under control and prevent infection of deeper cutaneous layers and of the entire organism [113]. Plant extracts enriched with secondary metabolites have been traditionally used in folk medicine to treat various human infectious diseases. The literature data have demonstrated that cinnamic acid and its esters possessed remarkable anti-fungal properties against the dermatophyte *Malassezia furfur* [153]. This effect depended on the inhibition of 17-beta hydroxysterol dehydrogenase (17βHSD), which is involved in the biosynthesis of steroids by the cell wall of fungi. The 17βHSD inhibition occurs when plant-derived actives bind to the active centre of the enzyme between nicotinamide moiety and tyrosine 212, thus blocking the enzyme activity [154]. The 17betaHSD is involved in the biosynthesis of steroid hormones in humans as well. Namely, the type III of 17betaHSD converts androstenedion into testosterone. In the prostate tumours, there is highly elevated expression of both enzymes 17betaHSD and 5 alpha reductase, an enzyme converting testosterone into dihydrotestosterone. The recent studies have shown that polyphenols of plant origin are efficient inhibitors of human 17betaHSD as well [155]. Low molecular weight lignins, polyphenol-containing polymers formed in solid cultures of *Lentinula edodes* (Berk) Pegler mycelia exhibited anti-viral effects towards human papilloma and hepatitis C viruses [156]. Major secondary metabolite parietin isolated from *Xanthoria parietina* L. lichen cultures has shown strong antibacterial activity against a wide range of bacterial strains, first of all, against *Staphylococcus aureus*, and antifungal effects against *Rhizoctonia solani*, in particular [157]. Recent studies of seven compounds isolated from *Lippia* species have confirmed very high anti-*Cryptococcus neoformans* activity of verbascoside, interpreted by the authors as a promising way for new selective anti-fungal verbascoside-containing topical drugs [158].

Several lines of evidence suggest that secondary metabolites produced by plant cells can combat skin infections in both ways, direct anti-microbial action and induction of intrinsic skin-located immune defence systems. Taken together, these mechanisms balance the normal microbiota pattern, thus eliminating risk of age-related skin pathologies, such as non-melanoma skin cancers, impaired wound healing, actinic keratosis (age spots), and skin infections.

## 7. Mechanisms Underlying Skin Ageing: Potential Targets for Active Ingredients of Meristem Plant Cultures

Skin is a universal innate defence to the organism against biotic and abiotic stresses. Being the outmost frontier between the internal organism and environment, it is continuously exposed to innumerable environmental or intentional man-made hazards. Apart from mechanical defence provided by low-penetrable and elastic cutaneous structure, four major lines of protection are constitutively expressed in the human skin: metabolic, redox, photochemical, and immune barriers [4,19]. All these defence mechanisms in the skin are interconnected and regulated by redox-dependent processes [20,145]. This resembles a situation in plants when the redox-dependent biosynthesis of protective/adaptive secondary metabolites begins under the challenge by either biotic or abiotic stresses. All four barrier mechanisms in the human skin are induced-upon-exposure by similar molecular pathways activated by transcription factors (Nrf2 and AhR) [19]. It has been hypothesised that inherited or acquired defects in one or several protective skin systems may generate disturbances in the others, alter their interactions, and ultimately lead to the development of distinct chronic skin pathologies as well as to chronologic (intrinsic) or premature (extrinsic/environment-induced) skin ageing. Several hypotheses of skin ageing are based on genetic, epigenetic, stem cells, immune, free radical, and metabolic components. All these essential components could be affected by secondary plants metabolites as it is extensively published elsewhere.

In general, cosmetic preparations should interact and positively affect at least some of the above mentioned skin ageing mechanisms, namely, (a) to protect from skin age-accelerating solar irradiation, to maintain normal redox balance in the skin; (b) to normalise skin microbiota thus preventing age-related deterioration of skin-located immune system; (c) to regulate epigenetic machine responsible for proper gene induction and suppression in the skin cells thus promoting skin regeneration; (d) to control metabolic processes in skin cells and extracellular matrix.

Our current knowledge brings us to the assumption that active secondary metabolites from meristem plant cells could finely tune all the above mentioned mechanisms of skin ageing hence promoting skin structure and functions characteristic of a young skin.

The combination of anti-bacterial, anti-inflammatory, and anti-androgen effects of purified verbascoside and of verbascoside rich plant extracts (*Camellia sinensis* (L.) Kuntze and *Commiphora mukul* (Hook. Ex Stocks)) seems to have perspectives in the development of cosmeceutical treatments for acne vulgaris [159]. Anti-replicative senescence effects via sirtuin-dependent mechanisms have been reported for meristem cells of *Leontopodium alpinum* L. and *Lippia citrobara* L. [30,160]. The topical application of meristem cells from *Syringa vulgaris* provided skin healing and UV-protection [2,37,41,106] as well as anti-inflammatory and anti-microbial effects [30,41].

Several cosmetic cell lines containing active ingredients of *Leontopodium alpinum* Cass., *Buddeleja davidii* Franch., *Centella asiatica* L., *Gardenia jasminoides* J.Ellis, and *Echinacea angustifolia* meristem cells have been recently developed and marketed claiming impressive and long-lasting cosmetic effects on skin appearance, due to rejuvenation of both skin cells and extracellular matrix. The implicated rejuvenation effects, based on the growing body of laboratory evidences, range from greatly enhanced skin hydration, elasticity, and sebum production to sparing of lipophilic skin antioxidants (vitamin E and coenzyme Q_10_), to reduced squalene and protein oxidation, and normalised skin microbiota pattern. According to commercial information, these age-reversing effects have become more evident when the meristem cell-containing cosmetics were used in combination with conventional procedures of skin rejuvenation, such as fractionation laser, collagen masks, fruit acid peeling, and radio frequency treatments. Nonetheless, reliable clinical data on the safety and efficacy of plant meristem cell-based cosmetic formulations are so far unfortunately unavailable.

## 8. Skin Rejuvenation by the Active Principles of Meristem Plant Cells: Background, Perspectives and Open Issues

In recent years, many speculations and aggressive advertisement have appeared on the “magic role” of plant stem cells in human skin rejuvenation. These misleading commercial claims are gone so far as to announce a “substitution of human stem cells by plant stem cells” or a “replenishment of growth factors by plant proteins”. These and other similar statements on plant stem cells efficacy, non-complying with international regulations requiring a proof of concept for cosmetic efficacy claims, are false and damaging both for cosmetic industries and customers alike.

Actually, plant cell cultures could be considered as a unique, sustainable, and valuable source of low-molecular weight active substances [33]—secondary metabolites (“green industries”). Given the vast experimental and clinical body of evidences showing numerous health effects of cultivated plant cell-derived secondary metabolites, their high bioavailability through the cutaneous barrier, the bio-compatibility to human cells, and the relatively well-defined mechanisms underlying the anti-ageing action, there seem to be brilliant perspectives for the use of these biotechnologically produced active ingredients in rejuvenation cosmetics [2,4,29,30]. Their excellent, multifaceted skin protection skills against solar UV, chronic cutaneous inflammation, carcinogenesis, and metabolic alterations, target many of the known-so-far molecular pathways leading to human skin ageing [29,30,34]. We hypothesise that the plant cell-derived actives for topical administration here described possess perfect redox-balancing properties. Our current knowledge suggests that a majority of processes defining skin cell growth, differentiation, and functioning is redox-regulated [37,84,113]. Hence plant cell-produced redox-active substances could substantially ameliorate not only redox, but also photochemical, immune, and metabolic barrier functions in the ageing skin [19].

Standing for this sustainable approach, biodiversity of Earth plant species, especially, extinguishing ones could be preserved; the load of environmental contaminants in future cosmetics could be significantly diminished, which would allow to marking them “fully biological”; and the spectrum as well as quality of active substances in plant-derived raw materials for cosmetic industry could be highly standardised [2,29].

However, this seemingly ideal source of active principles for cosmetic and skin care products currently presents several evident limitations, such as genetic and metabolic instability of the plant cell cultures, difficulties of their proper eliciting and up-scaling to industrial quantities, and so far also a comparatively high cost. With the development of modern combined biotechnologies using plant and microbial cultures, metabolic engineering, and improvement of plant cell cultivation/elicitation, more plant cell and tissue lines could become available for the stable cost-effective industrial production of their active metabolites. Advances in cosmetic technology, to develop appropriate vehicles able of protecting these active agents from oxidation and metabolic transformation, would increase their bioavailability through the cutaneous barrier [161,162,163]. New cosmetic forms would facilitate targeted delivery of the actives to definite skin layer/cell types. This would result in topical preparations with high and durable clinical/aesthetic efficacy. Pre-clinical and clinical safety and efficacy of these innovative cosmetic products with anti-age claims should be duly confirmed in reliable placebo-controlled clinical trials.

## Supplementary Materials

Supplementary File 1
