# Supplementary material for: Meristem Plant Cells as a Sustainable Source of Redox Actives for Skin Rejuvenation
Source: Biomolecules. 2017 May 12;7(2):40. doi: 10.3390/biom7020040 (PMC5485729; doi:10.3390/biom7020040)
Supplement: Supplementary File 1 [file biomolecules-07-00040-s001.pdf]

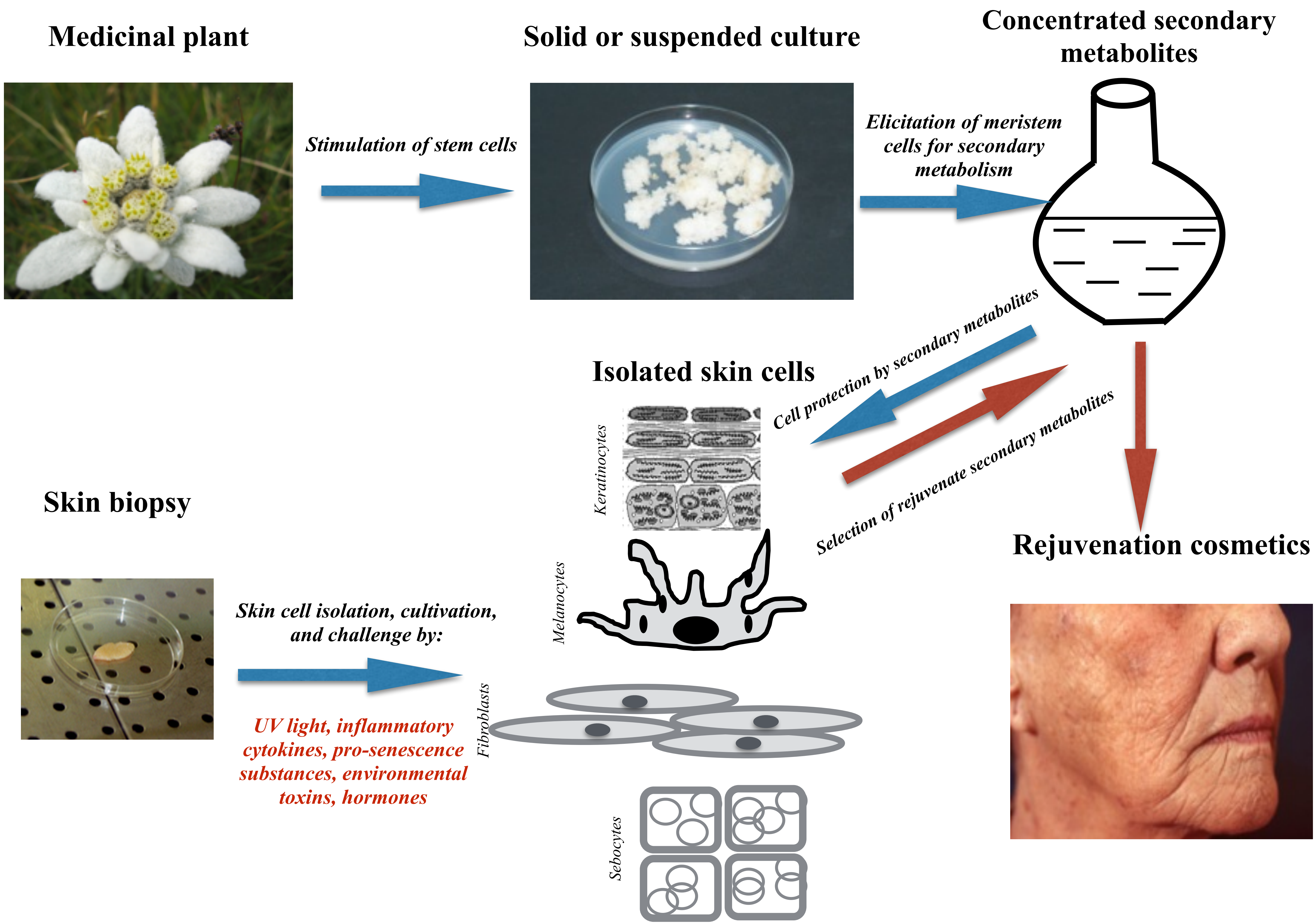

Medicinal plant
Solid or suspended culture
Concentrated secondary metabolites
Stimulation of stem cells
Elicitation of meristem cells for secondary metabolism
Isolated skin cells
Cell protection by secondary metabolites
Selection of rejuvenate secondary metabolites
Keratinocytes
Melanocytes
Fibroblasts
Sebocytes
Skin biopsy
Rejuvenation cosmetics
Skin cell isolation, cultivation, and challenge by:
UV light, inflammatory cytokines, pro-senescence substances, environmental toxins, hormones
